# Hilar Lymphadenectomy Is Associated With Improved Disease‐Free Survival in Pathologically N0 Non‐Small Cell Lung Cancer

**DOI:** 10.1002/wjs.70144

**Published:** 2025-10-14

**Authors:** Marco Chiappetta, Carolina Sassorossi, Filippo Lococo, Elisa Meacci, Maria Teresa Congedo, Jessica Evangelista, Annalisa Campanella, Giuseppe Calabrese, Alessia Senatore, Isabella Sperduti, Stefano Margaritora

**Affiliations:** ^1^ Thoracic Surgery Unit Magna Graecia University Catanzaro Italy; ^2^ Thoracic Surgery Unit Fondazione Policlinico Universitario A. Gemelli IRCCS Rome Italy; ^3^ Thoracic Surgery Unit Università Cattolica del Sacro Cuore Rome Italy; ^4^ Biostatistical Unit IRCCS Regina Elena National Cancer Institute Rome Italy

**Keywords:** lymph nodes, lymphadenectomy, NSCLC, sampling

## Abstract

**Background:**

Aim of this study is to evaluate the prognostic role of nodal parameter in early stage pathologically patients with N0 who underwent lobectomy and lymphadenectomy.

**Methods:**

Clinical and pathological characteristics of patients who underwent anatomical lung resection from 1/01/2010 to 31/12/2019 were reviewed and retrospectively analyzed. GGO and part‐solid tumors, MIA, AIS, more than 5 cm in size, with nodal and/or distant metastases, or receiving neoadjuvant treatment were excluded. Operatory and pathological report were reviewed to collect data on lymphadenectomy. The primary end‐point was disease‐free survival (DFS), calculated from surgery to recurrence appearance. Clinical/pathological characteristics and nodal parameters were associate to DFS using Kaplan–Meier curves. The log‐rank test was used to assess differences between subgroups. A multivariable model was built using Cox‐regression analysis, including variable resulting significant (*p* value < 0.05), at univariable analysis.

**Results:**

The final analysis was conducted on 487 patients. Most patients presented stage I tumor (82.4%). The mean number of resected nodes (#RN), resected N1 (#RN1) nodes, and resected N2 nodes (#RN2) resulted 9.5 ± 8.0, 3.4 ± 4.3, and 5.9 ± 4.4. The mean number of total resected stations (#RS), N1 resected stations (#RSN1), and N2 resected stations (#RSN2) resulted 2.5 ± 1.6, 1 ± 0.8, and 1.5 ± 1.2, respectively. During a mean follow‐up of 43 ± 28 months, a recurrence occurred in 137 (28.1%) patients. At univariable analysis, age < 70 years (*p* = 0.025), N1 lymphadenectomy (*p* = 0.019), #RSN1 ≥ 3 (*p* = 0.001), #RN ≥ 10 (*p* = 0.019), #RN1 ≥ 3 (*p* < 0.001), node sampling with more than 3 resected nodes (*p* = 0.049), at least 3 stations with 3 N1 nodes resected (*p* = 0.013), at least 3 stations resected with 10 lymphnodes, and 3N1 lymphnodes (*p* = 0.020) significantly correlated with improved DFS. Multivariable analysis confirmed as independent prognostic factor #RN1 ≥ 3 (*p* = 0.017; HR 1.782; and 95% CI: 1.107–2.867). Patients with #RN1 ≥ 3 presented a 5‐years DFS of 76.3% versus 57.8% of patients with #RN1 < 3 (*p* = 0.001).

**Conclusions:**

Hilar lymphadenectomy seems to significantly correlate with disease‐free survival in patients with pN0NSCLC and should be better defined in lymphadenectomy guidelines.

## Graphical abstract

Aim of this study is to evaluate the prognostic role of nodal parameter in early stage pathologically patients with N0 who underwent lobectomy and lymphadenectomy. Clinical and pathological characteristics of patients who underwent anatomical lung resection from 1/01/2010 to 31/12/2019 were reviewed and retrospectively analyzed. GGO and part‐solid tumors, MIA, AIS, more than 5 cm in size, with nodal and/or distant metastases, or receiving neoadjuvant treatment were excluded. Operatory and pathological report were reviewed to collect data on lymphadenectomy. The primary end‐point was disease‐free survival (DFS), calculated from surgery to recurrence appearance. Clinical/pathological characteristics and nodal parameters were associate to DFS using Kaplan–Meier curves. The log‐rank test was used to assess differences between subgroups. A multivariable model was built using Cox‐regression analysis, including variable resulting significant (*p* value < 0.05), at univariable analysis.

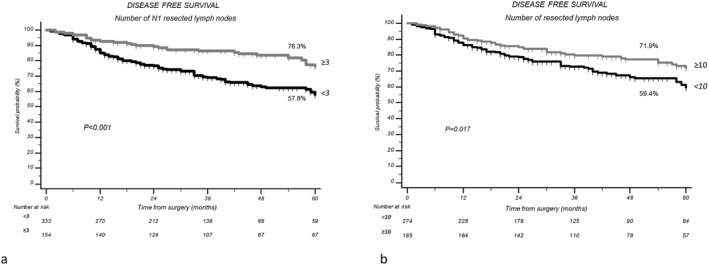

## 1 Introduction

Early stages non‐small cell lung cancer without nodal involvement presents a good survival rate, ranging between the 70% and 90% and it is associated with low recurrence risk [1, 2]. At this stage, surgery stands as the treatment of choice, consisting in anatomical resection and lymph node assessment [3, 4, 5], but which nodal parameter could define an adequate lymphadenectomy is still matter of debate. Indeed, different guidelines suggest the kind of nodal parameter that should be considered for adequate nodal assessment, mostly stations based, even if a count based definition is also possible. The European Society of Thoracic surgeons indicates a radical mediastinal nodal dissection and sampling of all hilar and mediastinal stations in every case [6], whereas other guideline advocates the importance of sampling at least one hilar and 3 mediastinal stations [3, 7]. Count‐based indications moved from the inclusion of at least 3 lymph nodes from hilar and mediastinal stations to at least 10 resected lymphnodes [8, 9]. The American College of Surgeons (ACS) Commission on Cancer (CoC) updated the indication from a count‐based sampling [9] to a station‐based sampling in 2022 [7], confirming the tendency to focus on the number of resected stations and not on the number of resected nodes, that may vary among patients and may present biases due to nodal fragmentation [10]. On the other hand, also a station based sampling may present some biases due to the amount of tissue resected, considering that especially mediastinal lymph nodes are included in mediastinal fat. As sampling definition implies, not all the fat tissue should be removed, so the number of included lymph nodes may be extremely various and not representative. As reported in the comment to the paper of Heiden and coworkers [11], the number of nodes in the nodal chain may variate, so, it could be possible to find only two nodes in a N2 station, resecting all the surrounding fat, or resecting only two representative nodes and still consider it as an appropriate sampling technique [12].

Moreover, data in literature reported that in case of nodal upstaging, it primarily involved the hilar stations [13, 14, 15] so the sampling of only one hilar station may be not sufficient. Another crucial point is that most of the station‐based guidelines did not report a minimum number of the resected mediastinal or hilar lymphnodes to confirm the adequate sampling.

In this study, we analyze the prognostic role of nodal parameters in terms of station‐based and count‐based sampling with the aim of identifying which parameters may better define an adequate lymphadenectomy in pathologically N0 early stage NSCLC.

## 2 Material and Methods

This is a single‐center study, held in our institution, Fondazione Policlinico A. Gemelli, IRCSS, in Rome, Italy. The study was approved by the Ethical committee of Regione Lazio Area 3 (Study ID: 6146).

Clinical and pathological characteristics of patients who underwent anatomical lung resection from 1/01/2010 to 31/12/2019 for NSCLC were reviewed and retrospectively analyzed. Pathological reports were reviewed and adapted to the 8th TNM staging system [2], while the following nodal parameters were collected:

Number of resected lymph nodes (#RN), number of resected stations (#RS), number of hilar resected lymph nodes (#RN1), number of hilar resected stations (#RSN1), number of mediastinal resected lymph nodes (#RN2), and number of mediastinal resected stations (#RSN2).

These parameters were also combined for a survival analysis considering groups based on number of resected N1, N2, or total lymph nodes and stations.

Categorization of #RN, #RN1, #RSN1, #RN2, and #RSN2 was decided according to literature data and available guidelines [3, 7, 8, 9, 10].

Inclusion and exclusion criteria were defined as follow:

Inclusion criteria were as follows:

- Pathological NSCLC
- Contrast CT scan and PET‐CT
- pT < 5 cm
- pN0
- Lymph node assessment
- Lobectomy
- Complete resection
- Follow‐up information

Exclusion criteria were as follows:

- Pure GGO
- Part‐solid tumors
- Minimally invasive adenocarcinoma
- Adenocarcinoma in situ
- Pleural invasion
- Distant metastases
- Neoadjuvant treatment

Lymphadenectomy was performed according the IASLC map [16], and hilar resected node were those harvested from hilar (station 10), interlobar (station 11), and lobar (station 12) lymph nodes.

The primary endpoint is disease‐free survival (DFS), calculated from surgery to recurrence appearance.

The secondary endpoint is to evaluate the recurrence site categorized as local or distant according to lymphadenectomy parameters.

Patients were classified free from disease when medical examination and follow‐up exams were deemed negative for suspected relapses or metastases.

Postoperative exams consisted in medical examinations, blood analyses, CT scans, and 18‐fluorodeoxyglucose positron emission tomography, when indicated, every 6 months for the first 2 years and every 6–12 months for 3 years thereafter.

Recurrences were classified as local if involved the operated lung and or hilar and mediastinal ipsilateral nodes; distant if located in the contralateral lung/pleural cavity or other sites.

### 2.1 Statistical Analysis

Descriptive statistics were used to summarize pertinent study information. The association between variables was tested using the Pearson’s chi‐squared test or Fisher's exact test when appropriate. The Kruskal–Wallis and the Mann–Whitney *U* (adjusted for multiple comparisons) nonparametric tests were used for variables with non‐Gaussian distributions. The hazard ratio and the confidence limits were estimated for each variable of interest using the Cox univariate model. Significance was defined at *p* < 0.05 level. At univariable analysis baseline demographic, clinical characteristics intraoperative, and pathological, such as sex, age, pathological stage, #RN, #RS, #RN1, #RSN1, #RN2, #RSN2, and combinations, were evaluated. A multivariate Cox proportional hazard regression model was also developed using stepwise regression (forward selection), including variables resulting significant in the univariate analyses and that were judged clinically relevant considering the aim of the study, reducing the presence of potentially redundant or extremely similar variables. Enter limit and remove limit were *p* = 0.10 and *p* = 0.15, respectively. Disease‐free survival (DFS) was calculated with the Kaplan–Meier product‐limit method from the date of the surgery to disease recurrence or death. If a patient did not experienced recurrence or death, DFS was censored at the time of the last visit. The log‐rank test was used to assess differences between subgroups. The SPSS (version 29.0; SPSS Inc., Chicago, IL), a licensed statistical program, was used for all analyses.

## 3 Results

The final analysis was conducted on 487 patients. Clinical and pathological results are reported in Table 1. Most patients presented stage I tumor (82.4%) and adenocarcinoma resulted the most frequent histology. The mean number of resected nodes (#RN), resected N1 (#RN1) nodes, and resected N2 nodes (#RN2) were 9.5 ± 8.0, 3.4 ± 4.3, and 5.9 ± 4.4, respectively. The mean number of total resected stations (#RS), N1 resected stations, (#RSN1) and N2 resected stations (#RSN2) were 2.5 ± 1.6, 1 ± 0.8, and 1.5 ± 1.2, respectively. Patients with more than 70 years received less resected lymphnodes in terms of mean RN2 (#RN2) and mean RN (#RN) compared to patients < 7 0 years: 3 (range 0–44) versus 5 (range 1–35; *p* = 0.033) and 7 (range 1–47) versus 8 (range 1–35; *p* = 0.029), respectively, whereas no differences considering the median number of N2 resected nodes (*p* = 0.246). Similarly, patients with pTstage Ib and IIa received a higher median number of #RN2 and #RN compared to stage Ia: 6 (range 1–44) versus 6 (range 1–18) versus 3 (range 1–38; *p* = 0.0035) and 12 (range 1–44) versus 14.5 (range 1–25) versus 7 (range 1–47; *p* < 0.0001).

**TABLE 1:** Clinical and pathological characteristics.

Variable	*N* (%) or mean
Sex	
Male	279 (57.1)
Female	208 (42.9)
Age	68 ± 9.6 years
Smoker	
Yes	245 (50.3)
No	229 (47.0)
Missing	13 (2.7)
Side	
Right	269 (55.2)
Left	218 (44.8)
Histology	
Squamous cell carcinoma	35 (7.1)
Adenocarcinoma	421 (86.5)
Carcinoid	21 (4.3)
Other	10 (2.1)
pT	
1a	66 (13.6)
1b	219 (45)
1c	116 (23.8)
2a	62 (12.7)
2b	24 (4.9)
pStage	
Ia	401 (82.3)
Ib	62 (12.7)
IIa	24 (4.9)
N1 lymphadenectomy	
Yes	352 (72.3)
No	127 (26.1)
Missing	8 (1.6)
Resected N1 stations	
0	127 (26.1)
1	234 (48.0)
> 1	118 (24.2)
Missing	8 (1.7)
Resected N2 stations	
< 3	367 (75.4)
≥ 3	111 (22.8)
Missing	9 (1.8)
Total resected stations	
< 3	232 (47.6)
≥ 3	234 (48.1)
Missing	21 (4.3)
Resected N1 lymphnodes	
< 3	333 (68.4)
≥ 3	154 (31.6)
Resected N2 lymphnodes	
< 6	266 (54.6)
≥ 6	191 (39.2)
Missing	30 (6.2)
Total resected lymphnodes	
< 10	274 (56.3)
≥ 10	185 (38.0)
Missing	28 (5.7)
Resected 1 N1 station, 3 N2 stations, and 10 nodes	
No	366 (75.2)
Yes	82 (16.8)
Missing	39 (8)
Resected 1 N1 station, 3 N2 stations, and 6 N2 nodes	
No	368 (75.6)
Yes	80 (16.4)
Missing	39 (8)
Resected 1 N1 station and 3 N2 stations	
No	352 (72.3)
Yes	96 (19.7)
Missing	39 (8)
Sampling total nodes	
0–3	124 (25.5)
4–9	150 (30.8)
≥ 10	185 (38.0)
Missing	28 (5.7)
Sampling N2 nodes	
0	102 (22.4)
1–5	157 (32.2)
≥ 6	191 (39.2)
Missing	30 (6.2)
Resected 1 N1 station, 3 N2 stations, and 3 N1 nodes	
No	389 (79.9)
Yes	59 (12.1)
Missing	39 (8)
Resected 3 stations and 3 N1 nodes	
No	311 (63.9)
Yes	135 (28.1)
Missing	39 (8)
Resected 3 stations and 10 nodes	
No	302 (62.0)
Yes	149 (30.6)
Missing	36 (7.4)
Resected 3 stations, 10 nodes, and 3 N1 nodes	
No	339 (69.6)
Yes	102 (20.9)
Missing	46 (9.5)

During a mean follow‐up of 43 ± 28 months, 5‐year overall survival resulted 90.6% (Figure S1). Recurrence occurred in 152 (31.2%) patients, resulting local in 118 (24.2%) patients, whereas distant recurrences occurred in 34 cases. At the univariable analysis, age < 70 years (*p* = 0.025), N1 lymphadenectomy (*p* = 0.019), #RSN1 ≥ 3 (*p* = 0.001), #RN ≥ 10 (*p* = 0.019), #RN1 ≥ 3 (*p* < 0.001), node sampling with more than 3 resected nodes (*p* = 0.049), at least 3 stations with 3 N1 nodes resected (*p* = 0.013), at least 3 stations resected with 10 lymph nodes and 3 N1 lymph nodes (*p* = 0.020) significantly correlated with improved DFS (Table 2).

**TABLE 2:** Univariable analysis.

Variable	*p* value	HR (95% CI)
Sex		
Male versus female	0.0.413	1.140 (0.833–1.559)
Age		
< 70 versus ≥ 70 years	0.025	1.427 (1.045–1.949)
Smoker		
Yes versus no	0.860	1.029 (0.749–1.413)
Side		
Right versus left	0.235	1.212 (0.883–1.664)
Histology	0.860	0.755 (0.200–2.850)
pT	0.322	0.854 (0.625–1.167)
pStage	0.598	
Ia versus IIa	485	1.311 (0.613–2.807))
Ib versus IIa	860	1.081 (0.454–2.576)
N1 lymphadenectomy		
Yes versus no	0.019	1.493 (1.069–2.086)
Resected N1 stations	0.001	
0 (ref.)		
> 1 versus 0	< 0.001	0.513 (0.358–0.736)
> 1 versus 1	0.006	0.539 (0.346–0.838)
Resected N2 stations		
< 3 versus ≥ 3	0.791	1.053 (0.722–1.532)
Total resected stations		
< 3 versus ≥ 3	0.116	1.288 (0.939–1.767)
Resected N1 lymphnodes		
< 3 versus ≥ 3	< 0.001	2.202 (1.480–3.289)
Resected N2 lymphnodes		
< 6 versus ≥ 6	0.333	1.178 (0.845–1.644)
Total resected lymphnodes		
< 10 versus ≥ 10	0.019	1.498 (1.070–2.098)
Resected 1 N1 station, 3 N2 stations, and 10 nodes		
No versus yes	0.475	1173 (0.757–1.818)
Resected 1 N1 station, 3 N2 stations, and 6 N2 nodes		
No versus yes	0.413	1.209 (0.768–1.904)
Resected 1 N1 station and 3 N2 stations		
No versus yes	0.572	1.127 (0.746–1.702)
Sampling total nodes	0.049	
0–3 (ref.)		
4–9	0.512	0.875 (0.588–1.303)
≥ 10	0.019	0.620 (0.415–0.924)
Sampling N2 nodes	0.579	
0 (ref.)		
1–5	0.585	1.123 (0.741–1.702)
≥ 6	0.297	1.219 (0.840–1.769)
Resected 1 N1 station, 3 N2 stations, and 3 N1 nodes		
No versus yes	0.175	1.448 (0.848–2.472)
Resected 3 stations and 3 N1 nodes		
No versus yes	0.013	1.607 (1.105–2.337)
Resected 3 stations and 10 nodes		
No versus yes	0.072	1.389 (0.971–1.986)
Resected 3 stations, 10 nodes, and 3 N1 nodes		
No versus yes	0.020	1.644 (1.080–2.501)

Multivariable analysis confirmed as independent prognostic factor #RN1 ≥ 3 (*p* = 0.017; HR 1.782; and 95% CI: 1.107–2.867; Table 3). A multivariable model, including also pStage, was also performed and it confirmed these results (Table S1).

**TABLE 3:** Multivariable analysis.

Variable	Multivariable *p* (HR; 95% CI)
Age	
< 70 versus ≥ 70 years	0.170 (0.792; 0.568–1.105)
N1 lymphadenectomy	
Yes versus no	0.841 (0.949; 0.568–1.585)
Resected N1 stations	0.434
0	Ref
1	0.510 (1.237; 0.656–2.332)
> 1	0.547 (0.867; 0.454–1.380)
N1 lymphadenectomy	
Yes versus no	0.841 (0.949; 0.568–1.585)
Resected N1 lymphnodes	
< 3 versus ≥ 3	0.017 (1.782; 1.107–2.867)
Total resected lymphnodes	
< 10 versus ≥ 10	0.456 (1.198; 0.746–1.924)
Resected 3 stations and 3 N1 nodes	
No versus yes	0.756 (1.123; 0.540–2.338)
Resected 3 stations, 10 nodes, and 3 N1 nodes	
No versus yes	0.775 (0.879; 0.364–2.123)

5‐year DFS resulted to be 62.5% for stage IA and 72.7% for stage IB.

**FIGURE 1:**
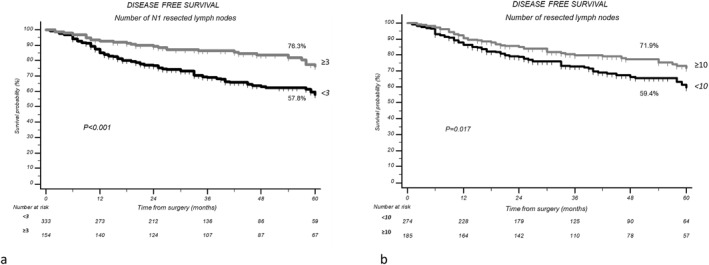
(a) Disease‐free survival according with number of resected N1 lymph nodes. (b) Disease‐free survival according with number of resected lymph nodes.

Patients with #RN1 ≥ 3 presented a 5‐year DFS of 76.3% versus 57.8% for patients with #RN1 < 3 (*p* = 0.001; Figure 1a). These results were confirmed also in pStage Ia (5 years DFS 62.5% and *p* = 0.004), pIb (5 years DFS 72.7% and *p* = 0.015), and pIIa (5 years DFS 62.9% and *p* = 0.015). Patients with #RN ≥ 10 presented a 5‐year DFS of 71.8% versus 59.4% of patients with #RN < 10 (*p* = 0.017; Figure 1b) whereas patients that presented 1 or more than 1 resected N1 station presented a significantly better DFS if compared to patients without N1 resected stations: 5‐year DFS 69.2% and 72.5% versus 52.1%, respectively (*p* < 0.001).

**FIGURE 2:**
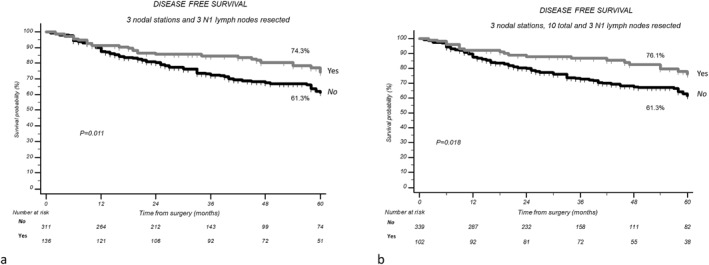
(a) Disease‐free survival according with at least 3 stations and 3 N1 nodes resected. (b) Disease‐free survival according with at least 3 stations resected, 10 lymph nodes, and 3 N1 lymph nodes resected.

Considering combinations of resected stations and lymph nodes, patients with at least 3 stations and 3 N1 nodes resected and patients with at least 3 stations resected, 10 lymph nodes, and 3 N1 lymph nodes presented a significantly improved DFS if compared to those without: 5YDFS 74.3% versus 61.3% (*p* = 0.011) and 76.1% versus 61.3% (*p* = 0.018; Figure 2a,b).

Lymph node parameters did not correlate with local or distant recurrence, whereas advanced *p* stage and sampling with more than 10 resected nodes were associated with a higher percentage of distant recurrences (Table S2).

## 4 Comment

In this study, we analyzed the nodal parameters that may define the adequate lymphadenectomy in early stage NSCLC that resulted N0 after surgical resection.

We found that in these patients, the dissection of at least 3 nodal stations and at least 10 lymph nodes significantly predict DFS, confirming previous literature studies suggesting the resection of 10 lymphnodes as a reliable prognosticator for survival in these patients [17, 18]. Moreover, we led a specific analysis considering the number of hilar or mediastinal lymphnode/stations, finding that in these patients, the extent of hilar assessment seems to play a significant role whereas the mediastinal lymphadenectomy did not. Indeed, the resection of 3 nodal stations and 3 hilar nodes or the resection of 3 stations, 10 nodes, and 3 N1 nodes significantly correlated with improved DFS.

In detail, hilar nodal assessment with the examination of at least three lymphnodes is an independent predictor for DFS, and the inclusion of this parameter in lymphadenectomy guidelines/recommendations may better define patient prognosis.

It is important to notice that this study aimed to identify prognostic factors in early stage patients with pN0, excluding those that presented pathologically confirmed N1 or N2 involvement for which the extent of mediastinal lymphadenectomy may be essential for upstaging [11, 19].

On the other hand, in patients resulting pathologically N0, it is important to evaluate the risk of residual disease due to inadequate nodal assessment, and this was the aim of this study.

Our results, underlining the importance of hilar assessment, are in agreement with the studies of dr Zhai et al. [20], who reported significant survival differences in patients with pT1T2N0 on the base of the number of analyzed N1 lymphnodes, comparing those with 0–5 versus > 5 analyzed lymphnodes. However, the authors dissected also the segmental and subsegmental nodes, which are not intraoperatively removed, whereas in our study, we focused on the stations usually dissected during surgery and not considered on the specimen. The identification of the minimum number of resected nodes remains an open and debated question for the need to give indication that then can be easily reproducible in the routinely clinical activity. Indeed, other studies reported significant differences considering a cut off of more than 10 N1 nodes [19] but most of them were intraparenchymal and may need time and resources to be examined, potentially reducing this application in daily practice. Moreover, especially in early stage tumors, a sampling strategy may be applicable and show similar results of more extended lymphadenectomy such as lobar specific or systematic dissections.

In our study, other nodal parameters lost the statistical significance at multivariable analysis, confirming that at this stage of disease, it is important to consider the closest nodal spreading ways, confirming the importance of hilar nodal assessment. Indeed, despite #RN resulted a significant factor in univariable analysis, the most important component seems to be the #RN1 included in the #RN

Our results suggest that the dissection of at least 3 lymph nodes is effective for prognosis prediction and consequently for defining and adequate hilar lymphadenectomy, and we also can underline the advantage to considered nodal stations that should be dissected during surgery and that do not request further analysis on the specimen.

Further larger or randomized studies are needed to validate our results, but, according to literature data [18, 19, 20, 21], hilar lymphadenectomy in terms of nodal stations or lymph nodes removed should be included in future guidelines to define adequate lymphadenectomy.

The need for a valid definition was underlined also by the IASLC, that implemented the UICC definitions about the completeness of resection introducing the uncertain resection concept, R(un) [22, 23]. This definition included consideration of the minimum parameters for lymphadenectomy, suggesting that intraoperative lymph node evaluation, which is less rigorous than systematic nodal dissection or lobe‐specific nodal dissection, does not ensure the removal of occult nodal metastases. Following this guideline, various studies demonstrated that this definition was associated with significantly better survival outcomes, with improved survival in patients undergoing adequate lymphadenectomy compared to those with R(un) resections. However, in some studies, the extent of lymphadenectomy was quite limited. Osarogiogbon et al. [24] reported a survival benefit in patients with R0 compared to R(un) based on lymphadenectomy extent, but only the 33% resulted R0 and about the 35% presented any mediastinal resected node.

It is quite clear that these approaches are not possible nowadays, but, on the other hand, more specific indications are needed about intraoperative nodal assessment, especially about hilar nodes evaluation. Indeed, the increased indications to segmentectomy opened new questions about the lymphadenectomy possibilities. In our study, considering the dissection of stations 10–12, we found that patients without this nodal evaluation presented a significantly shorter disease‐free survival, suggesting that occult nodal metastases may be present. During segmentectomy, the access to hilar, interlobar, and lobar lymph nodes may be limited due to absence of surgical exposure, with the risk of significantly reducing the hilar nodes evaluation.

Kawamoto et al. [25] reported involvement of hilar lymph nodes in 14% of stage I patients, regardless of tumor size, with a significant difference in the rate of hilar metastases between peripheral and central tumors (7.4% vs. 21.5% and *p* < 0.001). Although the authors did not provide details on the specific nodal stations or the number of lymph nodes removed, their study highlights the importance of hilar lymphadenectomy even in stage I patients, despite not finding significant differences in disease‐free survival.

Lee et al. [26] compared different groups based on adherence to the IASLC R and R (un) criteria, reporting that patients who underwent sublobar resection had a very limited nodal assessment. Although the choice of surgical approach appeared to be influenced by favorable histology and tumors smaller than 2 cm in approximately 70% of cases, it was evident that sublobar resections were associated with less extensive nodal evaluation. For these reasons, it will be important in the future to provide clear guidelines for segmentectomies, taking tumor size into account. An adequate lymphadenectomy should include specific recommendations for the hilar node assessment. From this perspective, guidelines based solely on the removal of one hilar station, without specifying the number of nodes to be resected, seem insufficient. As proposed by the IASLC [23], hilar sampling or lymphadenectomy should involve the removal of at least three lymph nodes.

Our study confirmed this recommendation, demonstrating a significant improvement in disease‐free survival (DFS) when hilar sampling was performed, particularly when the analysis included three lymph nodes. We also observed a significant DFS benefit when the number of N1 resected nodes was considered in a station‐based count, associating this with the removal of three resected stations. Additionally, including this parameter in a lymph node‐based count—considering three resected stations, 10 resected nodes, and nearly three N1 nodes—showed a positive impact on DFS. It is implicit that during segmentectomy, particularly with peripheral dissection of the bronchial tree, access to hilar or interlobar lymph nodes may be limited, increasing the risk of leaving occult nodal metastases behind. This possibility has been highlighted by other studies validating the R(un) definition, which is based on adequate lymphadenectomy. These studies demonstrated poorer survival outcomes for R(un) resections compared to R0 resections, likely due to missed hilar or mediastinal metastases, and the subsequent lack of appropriate adjuvant therapy [24, 27].

Other parameters will require further ad hoc analysis as it is crucial to balance the benefits of lymphadenectomy with the associated risks of complications. Indeed, nerve, lymphatic, or vascular injuries can occur, particularly during mediastinal lymphadenectomy [28, 29]. Identifying specific patient groups with an increased risk of nodal involvement will help avoid unnecessary extensive lymphadenectomy in low‐risk patients.

This study has some limitations due to its retrospective nature, which may introduce bias in patient selection and the extent of lymphadenectomy. Specifically, the decision regarding the extent of lymphadenectomy was made by the surgeon based on their experience, tumor location, size, and histology, rather than being predefined or standardized. To minimize this bias, we excluded cases with favorable histologies, such as minimally invasive adenocarcinoma (MIA) or adenocarcinoma in situ (AIS), as well as nodules with a ground‐glass opacity (GGO) appearance. These cases typically receive more limited lymphadenectomy due to their favorable prognosis and low risk of nodal spread. Another limitation is the potential for inaccurate nodal counting due to fragmentation. However, to address this issue, we implemented internal guidelines for lymphadenectomy and pathological analysis, which likely reduced this bias. Specifically, during sampling of the mediastinal stations, lymph nodes were dissected along with the surrounding mediastinal fat, minimizing the risk of manipulation and fragmentation. Additionally, pathologists counted the number of resected nodes based on visible lymph nodes at the hilum rather than including peripheral fragments.

On the other hand, the exclusion of these favorable histology may limit the extension of our indication to a significant part of tumors. Future studies will focus the risk of nodal involvement for these less aggressive subtypes, giving ad hoc indication for specific lymphadenectomy parameters.

Hilar lymphadenectomy is confirmed to be an important part of nodal assessment during surgery for non‐small cell lung cancer. The resection of at least 3 hilar nodes allows prognosis stratification and the assessment of the adequacy of lymphadenectomy.

## Author Contributions

**Marco Chiappetta:** conceptualization, investigation, methodology, writing – original draft, writing – review and editing, project administration. **Carolina Sassorossi:** conceptualization, project administration. **Filippo Lococo:** data curation, supervision. **Elisa Meacci:** resources. **Maria Teresa Congedo:** resources. **Jessica Evangelista:** resources. **Annalisa Campanella:** resources. **Giuseppe Calabrese:** resources. **Alessia Senatore:** resources. **Isabella Sperduti:** formal analysis. **Stefano Margaritora:** supervision, project administration.

## Ethics Statement

The study was approved by the Ethical committee of Regione Lazio Area 3 (Study ID: 6146).

## Consent

Informed consent was obtained from all individual participants included in the study.

## Conflicts of Interest

The authors declare no conflicts of interest.

## Supporting information


**Figure S1:** OS in the study cohort.

**Table S1:** Multivariable model n.2.

**Table S2:** Association between risk factors and local or distant recurrence.

## Data Availability

Data are properties and available among our Institution.
