# Supplementary figures and images for: Hilar Lymphadenectomy Is Associated With Improved Disease‐Free Survival in Pathologically N0 Non‐Small Cell Lung Cancer
Source: World J Surg. 2025 Oct 14;50(2):404–12. doi: 10.1002/wjs.70144 (PMC12904850; doi:10.1002/wjs.70144)

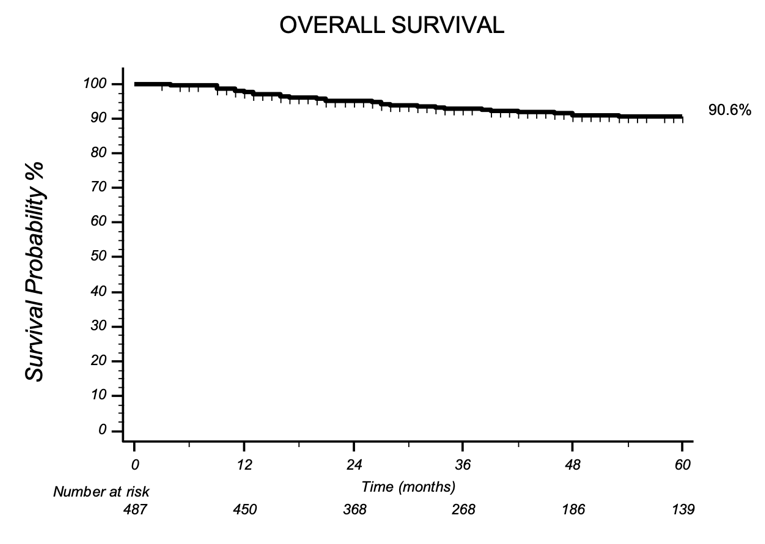


Supplemental figure

OS in the study cohort

Supplement: Supplementary file 1 — Figure S1: OS in the study cohort. [file WJS-50-404-s002.docx]
