# Supplementary material for: Hilar Lymphadenectomy Is Associated With Improved Disease‐Free Survival in Pathologically N0 Non‐Small Cell Lung Cancer
Source: World J Surg. 2025 Oct 14;50(2):404–12. doi: 10.1002/wjs.70144 (PMC12904850; doi:10.1002/wjs.70144)
Supplement: Supplementary file 2 — Table S1: Multivariable model n.2. [file WJS-50-404-s003.docx]

Supplemental table n1

Multivariable model n.2

| VARIABLE | Multivariable p (HR;96%CI) |
| --- | --- |
| Age  <70 vs ≥70 years | 0.092(0.1.327;0.955-1.843) |
| Number of N1 resected nodes | 0.930(0.981;0.647-1.488) |
| Number of resected nodes | 0.735(1.006;0.970-1.044) |
| pSTAGE | 0.754(1.0053;0.762-1.455) |
| Resected N1 lymphnodes  <3 vs ≥3 | 0.017(1.782;1.107-2.867) |
| Total resected lymphnodes  <10 vs ≥10 | 0.471(0.814;0.466-1.424) |
| Resected N1 stations | 0.393 (0.882;0.662-1.176) |
