# Supplementary material for: Hilar Lymphadenectomy Is Associated With Improved Disease‐Free Survival in Pathologically N0 Non‐Small Cell Lung Cancer
Source: World J Surg. 2025 Oct 14;50(2):404–12. doi: 10.1002/wjs.70144 (PMC12904850; doi:10.1002/wjs.70144)
Supplement: Supplementary file 3 — Table S2: Association between risk factors and local or distant recurrence. [file WJS-50-404-s001.docx]

Supplemental table 2.

Association between risk factors and local or distant recurrence.

| Variable | | Recurrence | |  |
| --- | --- | --- | --- | --- |
|  |  | **Local n (%)** | **Distant n (%)** | **p-value** |
| Age | < 70  ≥ 70 | 59 (50.0)  59 (50.0) | 13 (39.4)  20 (60.6) | 0.281 |
| Smoker | No  Yes | 53 (46.5)  61 (53.5) | 13 (39.4)  20 (60.6) | 0.470 |
| Side | Right  Left | 57 (50.4)  56 (49.6) | 22 (68.8)  10 (31.3) | 0.066 |
| Histology | Squamous  Adenocarcinoma  Carcinoid  Other | 5 (4.2)  107 (90.7)  4 (3.4)  2 (1.7) | 2 (5.9)  29 (85.3)  2 (5.9)  1 (2.9) | 0.834 |
| pStage | 1a  1b  2a | 103 (87.3)  11 (9.3)  4 (3.4) | 25 (73.5)  6 (17.6)  3 (8.8) | 0.051 |
| pStage (2) | 1  2 | 114 (96.6)  4 (3.4) | 31 (91.2)  3 (8.8) | 0.187 |
| Resected N1 stations | 0  1  >1 | 38 (33.0)  54 (47.0)  23 (20.0) | 13 (39.4)  17 (51.5)  3 (9.1) | 0.342 |
| Resected N2 stations | <3  ≥3 | 87 (75.7)  28 (24.3) | 29 (87.9)  4 (12.1) | 0.133 |
| Total resected stations | <3  ≥3 | 60 (52.6)  54 (47.4) | 22 (68.8)  10 (31.3) | 0.104 |
| Resected N1 lymph nodes | No  Yes | 89 (75.4)  29 (24.6) | 28 (82.4)  6 (17.6) | 0.398 |
| Total resected lymph nodes | <10  ≥10 | 68 (63.0)  40 (37.0) | 24 (75.0)  8 (25.0) | 0.208 |
| Resected N2 lymph nodes | <6  ≥6 | 64 (59.8)  43 (40.2) | 21 (67.7)  10 (32.3) | 0.424 |
| Resected 1 N1 station,3 N2 stations and 10 nodes | No  Yes | 90 (84.1)  17 (15.9) | 26 (86.7)  4 (13.3) | 0.999 |
| Resected 1 N1 station,3 N2 stations and 6 N2 nodes | No  Yes | 88 (82.2)  19 (17.8) | 27 (90.0)  3 (10.0) | 0.406 |
| Resected 1 N1 station,3 N2 stations | No  Yes | 86 (80.4)  21 (19.6) | 26 (86.7)  4 (13.3) | 0.430 |
| Sampling total nodes | 0-3  4-9  >9 | 29 (26.9)  39 (36.1)  40 (37.0) | 15 (46.9)  9 (28.1)  8 (25.0) | 0.050 |
| Sampling N2 nodes | 0  1-5  >=6 | 26 (24.3)  38 (35.5)  43 (40.2) | 10 (32.3)  11 (35.5)  49 (35.5) | 0.614 |
| Resected 1 N1 station,3 N2 stations and 3 N1nodes | No  Yes | 98 (91.6)  9 (8.4) | 27 (90.0)  3 (10.0) | 0.725 |
| Resected 3 stations and 3 N1 nodes | No  Yes | 83 (77.6)  24 (22.4) | 23 (76.7)  7 (23.3) | 0.917 |
| Resected 3 stations and 10 nodes | No  Yes | 76 (70.4)  32 (29.6) | 25 (80.6)  6 (19.4) | 0.258 |
| Resected 3 stations, 10 nodes and 3 N1 nodes | No  yes | 87 (82.9)  18 (17.1) | 25 (83.3)  5 (16.7) | 0.951 |
